# *CATION AMINO ACID TRANSPORTER1* encodes an arginine transporter whose expression is influenced by daylength and *Photoperiod-1*

**DOI:** 10.1093/aob/mcaf206

**Published:** 2025-09-12

**Authors:** Marianna Pasquariello, Yue Qu, Adam Gauley, Adam Bentham, Laura E Dixon, Scott A Boden

**Affiliations:** Department of Crop Genetics, John Innes Centre, Norwich Research Park, Norwich NR4 7UH, UK; School of Agriculture, Food and Wine, Waite Research Institute, University of Adelaide, Glen Osmond, SA 5064, Australia; Department of Crop Genetics, John Innes Centre, Norwich Research Park, Norwich NR4 7UH, UK; Department of Agronomy and Plant Science, Agrifood and Biosciences Institute, Belfast BT6 9SH, UK; Department of Crop Genetics, John Innes Centre, Norwich Research Park, Norwich NR4 7UH, UK; Centre for Programmable Biological Matter, Department of Biosciences, Durham University, Durham DH1 3LE, UK; Department of Crop Genetics, John Innes Centre, Norwich Research Park, Norwich NR4 7UH, UK; Genebank Department, Leibniz Institute of Plant Genetics and Crop Plant Research, Seeland D-06466, Germany; Department of Crop Genetics, John Innes Centre, Norwich Research Park, Norwich NR4 7UH, UK; School of Agriculture, Food and Wine, Waite Research Institute, University of Adelaide, Glen Osmond, SA 5064, Australia

**Keywords:** Amino acids, arginine, transporter, photoperiod, wheat

## Abstract

**Background and Aims:**

Flowering and inflorescence development of wheat involve coordination of organ development, tissue growth, and remobilization of nutrients from source to sink tissue. *Photoperiod-1* (*Ppd-1*) is a major flowering gene that regulates diverse genetic pathways during inflorescence development, including those that determine spikelet and floret formation; however, it is not yet known if Ppd-1 influences the expression of genes encoding proteins that transport nutrients.

**Methods:**

Here, we used transcriptome data from near-isogenic lines that contain variant *Ppd-1* alleles to show that Ppd-1 is required for expression of a cation amino acid transporter, *CATION AMINO ACID TRANSPORTER1* (*CAT1*), in developing inflorescences and leaves.

**Results:**

The influence of Ppd-1 on *CAT1* expression is supported by *CAT1* activity being photoperiod-responsive, with *CAT1* transcripts absent in short days and accumulating as daylengths extend. Functional analysis using *Xenopus* oocytes supports a role for *CAT1* as a transporter of cationic amino acids, including arginine, and characterization of mutant lines lacking functional CAT-D1 and CAT-B1 show that CAT1 influences amino acid levels, root growth and spikelet development.

**Conclusions:**

Our results indicate Ppd-1 has a broader role during wheat reproductive development by affecting the expression of amino acid transporters such as *CAT1*, which encodes an arginine transporter that influences development and growth of source and sink tissues.

## INTRODUCTION

Flowering is a critical stage in a plant’s life cycle that responds to changes in daylength and temperature to align fertilization and seed development with favourable environmental conditions (Fjellheim *et al.*, 2014). Wheat is a long-day plant that flowers as daylengths extend during the transition from winter to spring, and flowering begins with the formation of a spike inflorescence that produces spikelets with grain-bearing florets (Guo *et al.*, 2017; Gauley and Boden, 2021). A vital aspect of flowering, from the initiation of inflorescence development through to fertilization and grain development, involves the remobilization of carbon- and nitrogen-based assimilates from source to sink tissue, which significantly influences grain number and quality (Reynolds *et al.*, 2022; Carrera *et al.*, 2024).

The predominant form of nitrogen transported in plants is amino acids, and their movement from source to sink tissue occurs through the phloem and xylem (Pate, 1973; Ortiz-Lopez *et al.*, 2000; Rentsch *et al.*, 2007). Amino acid transporters facilitate the loading and unloading of amino acids during their transport by mediating movement across cell membranes (Ortiz-Lopez *et al.*, 2000; Rentsch *et al.*, 2007; Tegeder and Rentsch, 2010). Two broad groups of plant amino acid transporters include the amino acid/auxin permeases (AAAPs) and the amino acid polyamine and choline transporters (APCs) (Rentsch *et al.*, 2007; Pratelli and Pilot, 2014). The AAAP subfamily comprises five subgroups, including the amino acid permeases, lysine–histidine transporters, proline transporters, γ-aminobutyric acid (GABA) transporters, aromatic and neutral amino acid transporters and auxin transporters. The smaller group of APC transporters comprises three subfamilies, including cation amino acid transporters (CATs), L-type amino acid transporters (LATs) and bidirectional acid transporters (BATs), which help with the uptake of amino acids by roots and mesophyll cells and the supply of amino acids to sink tissues (Su *et al.*, 2004; Hammes *et al.*, 2006; Tegeder and Rentsch, 2010; Yang *et al.*, 2014, 2015). The CATs, which are the focus of this study, influence a range of physiological processes including seedling and root growth, flowering, senescence and responses to abiotic stress in *Arabidopsis thaliana* and poplar (Su *et al.*, 2004; Hammes *et al.*, 2006; Couturier *et al.*, 2010; Yang *et al.*, 2014, 2015). The CATs have been primarily characterized as transporters of cationic amino acids, including arginine, lysine and histidine, while other members, such as CAT6 of *Arabidopsis*, display broader substrate specificity and transport large and unchained amino acids (Frommer *et al.*, 1995; Su *et al.*, 2004; Hammes *et al.*, 2006; Yang *et al.*, 2014; Jungnickel *et al.*, 2018). The identity and expression of CAT-encoding genes have been investigated in multiple species, including *Arabidopsis*, wheat, maize, rice, cotton, soybean, poplar and common ice plant, and in many cases the genes are expressed in source and sink tissue, consistent with a potential role in remobilizing nitrogen-based assimilates during reproductive development (Popova *et al.*, 2003; Su *et al.*, 2004; Hammes *et al.*, 2006; Yang *et al.*, 2014; Cheng *et al.*, 2016; Wan *et al.*, 2017; Gao *et al.*, 2024; Islam *et al.*, 2024).

The spike is a vital sink tissue in wheat and assimilate distribution to it contributes substantially to yield, with the spike dry weight at anthesis correlating significantly with grain number (Fischer, 2007; Reynolds *et al.*, 2022; Slafer *et al.*, 2023). The distribution of nitrogen- and carbon-based assimilates to the spike supports spikelet and floret primordium formation, spike and rachis growth, floret fertility and survival, and grain filling, and these traits respond to changes in nutrient supply (Langer and Liew, 1973; McDonald, 1992; Abbate *et al.*, 1995; Miralles, *et al.*, 2000; Fischer, 2007; Slafer *et al.*, 2014; Dixon *et al.*, 2022; Reynolds *et al.*, 2022). To investigate molecular processes that contribute to these traits, we studied the influence of genetic variation for *Photoperiod-1* (*Ppd-1*) on the transcriptome of the developing spike (Gauley *et al.*, 2024). *Ppd-1* is the major gene controlling photoperiod responsiveness in wheat and it helps regulate flowering time and traits affected by altered assimilate supply, including spikelet architecture, floret fertility and grain number (Beales *et al.*, 2007; Boden *et al.*, 2015; Ochagavia *et al.*, 2018; Perez-Gianmarco *et al.*, 2019; Gauley and Boden, 2021; Gauley *et al.*, 2024). Our analysis of photoperiod-sensitive wheat, relative to near-isogenic lines that contain either the early-flowering photoperiod-insensitive allele (*Ppd-D1a*) or the late-flowering null alleles for *Ppd-1* on all three genomes (*ppd-1*), showed that *Ppd-1* strongly influences the transcriptome of a spike during early developmental stages (Shaw *et al.*, 2012, 2013; Gauley *et al.*, 2024). The transcriptome analysis showed that absence of *Ppd-1* function substantially altered gene activity in the developing inflorescence, with ∼30 000 transcripts being differentially expressed in *ppd-1* relative to wild-type (Gauley *et al.*, 2024). Our functional characterization of genes that are differentially expressed in the *ppd-1* mutant focused on transcripts that are upregulated in *ppd-1* relative to wild-type; however, our analysis also showed that multiple genes were downregulated or suppressed in the *ppd-1* mutant (Gauley *et al.*, 2024).

In this study, we analysed the effect of *Ppd-1* and daylength on the expression of *CAT* genes and show that *CAT1* encodes an arginine-transporting protein whose expression depends on Ppd-1 and is photoperiod-responsive. Our mutant analysis indicates that CAT1 influences the growth and development of source- and sink-related tissues in wheat, including spikes and roots. These data highlight a potential role for *Ppd-1* in controlling the expression of proteins involved in the distribution of nitrogen-based assimilates from source to sink tissue.

## MATERIALS AND METHODS

### Plant materials

Hexaploid wheat genotypes (*Triticum aestivum*) used here included the wild-type photoperiod-sensitive cv. ‘Paragon’ and ‘Paragon’ near-isogenic lines (NILs) containing either the *Ppd-D1a* photoperiod-insensitive allele, null *ppd-1* alleles on the A, B and D genomes, or a null allele of *FT-B1* (Dixon *et al.*, 2018). Other genotypes included the wild-type cv. ‘Cadenza’ (also photoperiod-sensitive), and *CAD1865* and *CAD0972* obtained from the hexaploid wheat TILLING population that contain nonsense mutations in *CAT-D1* and *CAT-B1*, respectively (Krasileva *et al.*, 2017). The ‘Paragon’ NILs containing the *Ppd-D1a* photoperiod-insensitive allele and the null *ppd-1* alleles are the same lines generated and used in previous studies (Shaw *et al.*, 2012, 2013; Gauley and Boden, 2021; Gauley *et al.*, 2024). The *cat-D1_m1* used here was a BC_2_F_3_ line generated from a cross between *CAD1865* and ‘Cadenza’, produced to reduce background mutations, and the *cat1_m1* mutants were derived from a cross between *CAD1865* and *CAD0972*, which had each been backcrossed to ‘Cadenza’. The mutants were named according to wheat gene nomenclature guidelines (Boden *et al.*, 2023).

### Growth conditions

The cultivar ‘Paragon’ and the two *Ppd-1* NILs (described in Gauley and Boden, 2021) were grown at field sites based at Church Farm, John Innes Centre, Bawburgh, Norfolk, UK (52°62'25.7“*N*, 1°21'83.2”E) in 1-m^2^ plots, as described previously (Gauley and Boden, 2021). For experiments performed under controlled environment conditions, ‘Paragon’, *ft-B1, cat-D1_m1* and *cat1_m1* plants were grown in growth cabinets under short-day (8 h/16 h light/dark) or long-day (16 h/8 h light/dark) photoperiods at 300 µmol m^2^ s^−1^ (using Plantastar 400-W HQI bulbs [Osram] and Maxim 60-W tungsten bulbs) and 20 °C/15 °C (day/night) temperatures.

### RNA extractions and quantitative real-time PCR

Samples used for the RNA extraction and cDNA synthesis included the following tissues: (1) developing inflorescences of the vegetative (VG), double ridge (DR), lemma primordium (LP) and terminal spikelet (TS) stages; (2) leaves from plants grown under field conditions, with samples collected every 3–4 h during the day and night, and at dusk (sunset) when the days were 10, 11, 12 or 13 h long; and (3) leaves (emerging, 4th leaf stage and flag leaf), stem internodes at the late terminal spikelet stage from glasshouse-grown plants, and roots from plants grown on filter paper. Leaf and stem RNA extractions were performed using the Spectrum Plant Total RNA Kit (Sigma–Aldrich). RNA extractions from developing inflorescences were performed using the RNeasy Plant Mini Kit (Qiagen). cDNA synthesis and reverse transcriptase quantitative PCR (RT–qPCR) were performed as described previously, using a LightCycler480 Instrument II (Roche Life Science) (Gauley and Boden, 2021). Candidate gene expression from leaf and inflorescence was normalized using TraesCS6D02G145100, which we previously verified to be stably expressed in leaves, inflorescences and roots across different photoperiods (Gauley and Boden, 2021). RT–qPCR data are the average of at least three biological replicates and two technical replicates per reaction.

### Protein structure prediction of CAT-D1

The structure of *Ta*CAT-D1 protein was predicted using AlphaFold2 as implemented via ColabFold v1.5.5 (Jumper *et al.*, 2021; Mirdita *et al.*, 2022) and visualized using ChimeraX (v1.7.1) (Meng *et al.*, 2023). The predicted local-distance difference test (pLDDT) scores were used to assess the confidence of the structure, with residues coloured according to their pLDDT values. Residues with a pLDDT >90 were considered highly confident. The AF2-predicted structure of CAT-D1 was superimposed on the crystal structure of the cationic amino acid transporter (*Gk*ApcT) from *Geobacillus kaustophilus* (PDB: 6F34) (Jungnickel *et al.*, 2018) using the ChimeraX Matchmaker function (Meng *et al.*, 2023).

### *Xenopus* oocyte electrophysiology

*TaCAT-D1* was cloned into pOO2-GW using Gateway cloning technology, and the resulting plasmid was linearized using HpaI (New England Biolabs) and purified by ethanol precipitation. *CAT-D1* cRNA was synthesized from the linearized plasmid using the HiScribe SP6 RNA synthesis kit (New England Biolabs), and RNA was recovered and purified using lithium chloride precipitation. We injected 46 nL/23 ng of cRNA or equal volumes of RNase-free water into *Xenopus laevis* oocytes with a Nanoject II microinjector (Drummond Scientific). Oocytes were incubated for 48 h in ND100 Calcium Ringer’s solution (100 mm NaCl, 2 mm KCl, 1 mm MgCl_2_, 5 mm HEPES, 1.8 mm CaCl_2,_ pH 7). Electrophysiology was done as previously described (Roy *et al.*, 2008). Membrane currents and resting membrane potentials were recorded in different solutions including ND100 (pH 7), ND100 (pH 5.5), ND100 (pH 5.5) + 10, 20 or 50 mM l*-*arginine, and ND100 (pH 5.5) + 10 mM l*-*leucine.

### Amino acid quantification

Quantification of amino acids was performed on leaves from plants grown until the fourth- or fifth-leaf stage. Plants were grown under long daylengths for analysis of wild-type (cv. ‘Cadenza’) and *cat-D1_m1* and *cat1_m1* mutants. For the photoperiod shift experiment, seedlings were grown under short-day photoperiods (8 h light/16 h dark) until the four-leaf stage, and then half of the plants were shifted to long-day photoperiods (16 h/8 h) for 5 d before leaf samples were collected. Amino acid quantification was performed using nuclear magnetic resonance spectroscopy, as described previously, with five or six biological replicates performed per condition (Dixon *et al.*, 2022).

### Phenotype analyses

For root growth assays, wild-type cv. ‘Cadenza’ and *cat1_m1* seeds were geminated on Whatman filter paper soaked with sterile water and stratified at 4 °C for 3 d. Seeds that displayed an emerging radicle were selected for further analysis. Molten agar (0.8 % w/v, sterilized water) with either 1.25 or 2.5 mM l*-*arginine or water as a control was poured into 24 × 24-cm bioassay growth plates, and the medium was covered with Whatman grade 1 filter paper soaked with sterile water or l*-*arginine solution at the appropriate concentration (1.25 or 2.5 mm). Germinated seeds of each genotype were transferred to each dish (four replicates per genotype per plate), with three replicates performed for each condition. The seeds were incubated in a controlled environment room under a long-day photoperiod (16 h/8 h day/night) at 20/16 °C day/night temperature. The covers of each dish were left slightly ajar to facilitate shoot growth, and dishes were positioned at an angle of ∼40° and partially submerged with sterile water to avoid medium desiccation. Images of roots were taken 5 and 7 d after germination, and root length was measured using Fiji software (Schindelin *et al.*, 2012).

For the analysis of flowering time and spike architecture, plants were grown under a long-day photoperiod (16 h/8 h day/night) at 20/16 °C day/night temperature. Flowering time was determined as the number of days post-germination for the inflorescence of the main shoot to emerge from the boot (six replicates per genotype), and spikelet number was recorded for three inflorescences (main shoot and two tillers) from each plant (six replicates per genotype).

### Statistical analysis

The data shown in the figures are mean ± standard error of the mean. In boxplots, the box is bound by the lower and upper quartiles, the central bar represents the median, and whiskers indicate the minimum and maximum values of the biological replicates. Pairwise differences between genotypes or conditions were analysed using a two-tailed Student’s *t*-test; the *cat-D1_m1* and *cat1_m1* mutants were compared with wild-type independently and data were combined in the same graphs to simplify the presentation. All data analyses were carried out using R (v4.0.0).

## RESULTS

### Analysis of *CAT* gene expression in developing inflorescences

**Fig. 1.**
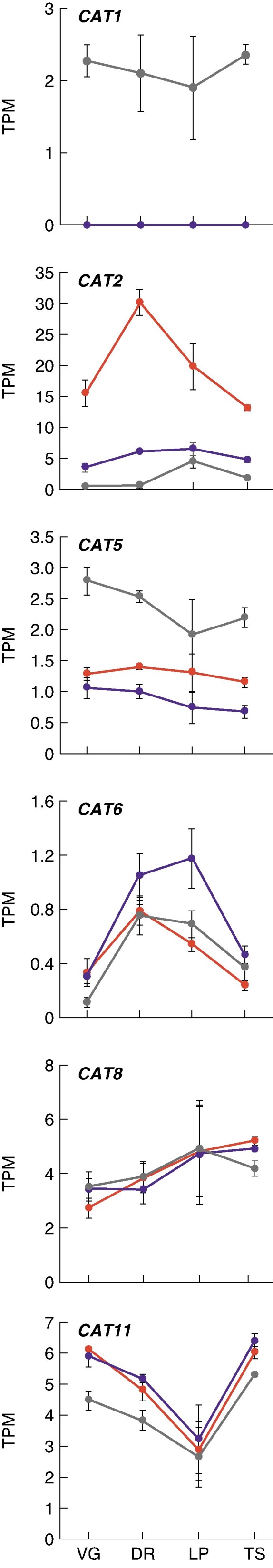
Transcript analysis of *CAT* genes during early inflorescence development. Analysis of *CAT* transcripts for *CAT1*, *CAT2*, *CAT5*, *CAT6*, *CAT8* and *CAT11* homeologues from the A (red), B (purple) and D (grey) subgenomes during early inflorescence development. The stages include: vegetative (VG), double ridge (DR), lemma primordium (LP) and terminal spikelet (TS). Transcript levels are shown as the average TPM of three biological replicates ± standard error of the mean.

Our analysis of CAT expression and function began with examining transcript levels of *CAT* genes during early inflorescence development, specifically the VG, DR, LP and TS stages (Supplementary Data Table S1). Our RNA-seq transcriptome analysis of wild-type wheat (cv. ‘Paragon’) showed that *CAT1*, *CAT2*, *CAT5*, *CAT6*, *CAT8* and *CAT11* are expressed during early inflorescence development (Fig. 1), with no transcripts detected for the remaining genes (i.e. *CAT3*, *CAT4*, *CAT7*, *CAT9* and *CAT10*) (Gauley *et al.*, 2024). For *CAT1*, transcripts were detected for the D genome homeologue (*CAT-D1*) at constant levels for each of the four stages, but not for the A or B genome homeologues (*CAT-A1*, *CAT-B1*; Fig. 1). All three homeologues were expressed for *CAT2*, *CAT5*, *CAT6*, *CAT8* and *CAT11*, which displayed different patterns during the four stages (Fig. 1). *CAT2* was the most highly expressed CAT-encoding gene, with *CAT-A2* transcripts being the most abundant homeologue, which peaked at DR. *CAT11* was also expressed strongly, with transcripts of all three homeologs decreasing at LP relative to the other three stages. *CAT8* was expressed during all four stages, with transcripts for all three homeologues increasing gradually from the VG to the TS stage. *CAT5* expression was relatively constant across all four stages, with *CAT-D5* transcripts being higher than the other two homeologues, while *CAT6* had the lowest expression, with transcripts for all three homeologues rising above the 0.5 transcripts per million (TPM) threshold only at the DR and LP stages. We further investigated the A and B homeologs of *CAT1* to determine if the undetected transcripts were caused by absence of homeologues in the A and B subgenomes. *CAT1* sequence was absent in the A subgenome of cv. ‘Paragon’ and other sequenced bread wheat genomes, consistent with previous reports (Wan *et al.*, 2017; Tian *et al.*, 2020; Walkowiak *et al.*, 2020). *CAT1* sequence was also absent in the A subgenome of tetraploid wheat and the genome of *Triticum uratu*, which is the A genome progenitor (Ling *et al.*, 2018; Walkowiak *et al.*, 2020). Together, these results indicate there is no copy of *CAT1* on the A subgenome, and its absence predates the genome hybridization events of tetraploid and hexaploid wheat. For *CAT-B1*, *CAT1* does exist in the B genome of cv. ‘Paragon’ and other sequenced genomes of bread wheat; however, qRT–PCR analysis confirmed that the gene is not expressed in the inflorescence or leaf tissue of cv. ‘Paragon’.

### *CAT1* expression is influenced by *Ppd-1* and is photoperiod-responsive

**Fig. 2.**
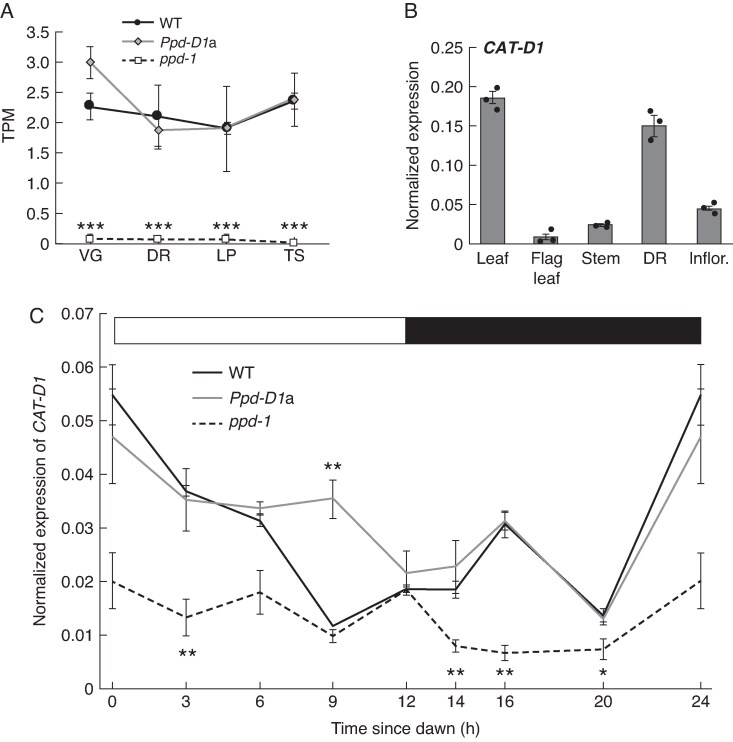
*CAT-D1* expression is dependent on *Ppd-1* function. (A) *CAT-D1* transcript levels are significantly lower during early inflorescence development in *ppd-1* null lines (dashed line), relative to wild-type (black line) and near-isogenic lines that contain photoperiod-insensitive alleles of *Ppd-D1* (*Ppd-D1a*; grey line). Samples were collected from field grown plants (Gauley *et al.*, 2024). (B) *CAT-D1* transcripts are detected in young leaves, immature inflorescences (DR, double ridge) and at lower levels in the flag leaf, stem and emerging inflorescence (Inflor.) in plants grown under long daylengths. (C) Diel expression analysis of *CAT-D1* in leaves of wild-type (black), photoperiod-insensitive (*Ppd-D1a*; grey) and *ppd-1* null (dashed line) lines under day neutral photoperiods (12 h light/12 h dark; represented by white and black bars). Data are average ±standard error of the mean of three biological replicates. In (A) the data are expressed as TPM and in (B) and (C) transcript values are shown as normalized expression values.

Following identification of *CAT* genes that are expressed in the developing inflorescence, we asked if their transcript levels are influenced by *Photoperiod-1* (*Ppd-1*), which is a major regulator of flowering and spikelet development. To test this, we examined transcripts for each CAT-encoding gene in developing inflorescences of NILs that contain either a photoperiod-insensitive allele of *Ppd-D1* (*Ppd-D1a*) or null alleles for *Ppd-1* on all three genomes (*ppd-1*) (Shaw *et al.*, 2012, 2013). The *Ppd-D1a* allele did not significantly modify transcript levels of *CAT* genes expressed in developing inflorescences, nor did it activate the expression of *CAT* genes that were silent in wild-type (Supplementary Data Fig. S1). We did, however, find that *CAT-D1* transcripts were absent in the *ppd-1* nulls during all four stages, indicating Ppd-1 function is required to promote *CAT-D1* expression (Fig. 2A). Interestingly, the *Ppd-1-*dependence for *CAT-D1* expression was not shared by other *CAT* genes, with *CAT-A2*, *-B2*, *-D2*, *-D6* and *-D8* transcripts being significantly higher at certain developmental stages relative to wild-type, and while *CAT-A11* and *-B11* transcripts were significantly lower in *ppd-1* at the double ridge stage, transcripts were detected at comparable levels at other stages (Supplementary Data Fig. S1). Transcripts of *CAT5*, *CAT-A6*, *CAT-B6*, *CAT8* and *CAT-D11* were not significantly different at any stage in the *ppd-1* null relative to wild-type (Supplementary Data Fig. S1). To further investigate the *Ppd-1*-dependent regulation of *CAT1* expression, we asked if *CAT-D1* transcripts are modified in leaves of the *Ppd-D1a* and *ppd-1* NILs relative to wild-type; tissue-specific analysis showed *CAT-D1* is expressed similarly in leaves as in the developing inflorescence, but transcripts are significantly lower in the stem, flag leaf and mature inflorescence at booting (Fig. 2B). We analysed *CAT-D1* expression in leaves of plants grown under day-neutral photoperiods (12 h light/12 h dark) when the inflorescence meristem is undergoing the vegetative-to-reproductive transition (Fig. 2C). Our analysis showed that *CAT-D1* transcripts were significantly reduced in *ppd-1* null lines at multiple time points relative to wild-type and photoperiod-insensitive plants, supporting a role for *Ppd-1* in regulating *CAT1* expression. In wild-type, *CAT-D1* transcripts peaked at dawn and decreased during the day, before displaying a second peak 4 h after dusk. *CAT-D1* displayed a similar diel expression pattern in the photoperiod-insensitive lines, except for 9 h after dawn, when transcripts were significantly higher relative to wild-type. This analysis indicates that *Ppd-1* affects the expression of *CAT1* in developing inflorescence and leaves.

**Fig. 3.**
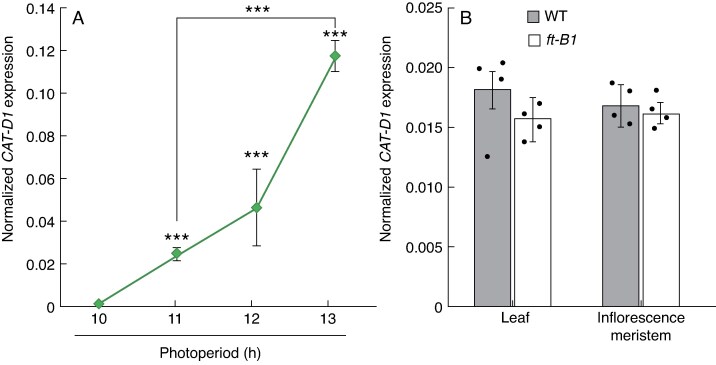
*CAT-D1* expression is induced by long-day photoperiods. (A) *CAT-D1* is not expressed in leaves under short-day photoperiods (e.g. 10 h), and is induced as daylength increases. Values are the expression of *CAT-D1* at the time point of 6 h after dawn. (B) Expression of *CAT-D1* under long-day photoperiods in leaves and the inflorescence meristem is independent of *FT-B1*. Data are the average ± standard error of the mean of three or four biological replicates. ***P < 0.001. WT, wild-type.

Given *Ppd-1* is a major regulator of photoperiod responsiveness in wheat, we hypothesized that *CAT-D1* expression is influenced by changes in daylength. To test this hypothesis, we measured *CAT-D1* transcripts during the day and night in plants grown under different photoperiods (Fig. 3A; Supplementary Data Fig. S2). We did not detect *CAT-D1* transcripts in plants grown under 8–10 h photoperiods, demonstrating that *CAT-D1* is not expressed under short-day photoperiods. However, *CAT-D1* transcripts were detected at 11 h at both the day and night time points, and they increased significantly as daylengths extended to 12 and 13 h, indicating *CAT1* expression is induced by long-day photoperiods and is daylength-responsive.

An important role for *Ppd-1* in wheat is to regulate the photoperiod-dependent expression of *FLOWERING LOCUS T1* (*FT1*), which is a central integrator of flowering. Photoperiod-insensitive lines induce *FT1* expression and flower earlier under short daylengths relative to photoperiod-sensitive genotypes, while *FT1* expression is significantly lower in *ppd-1* plants (Beales *et al.*, 2007; Shaw *et al.*, 2012; Shaw *et al.*, 2013; Boden *et al.*, 2015; Gauley and Boden, 2021). We therefore asked if there is an interaction between *FT1* and *CAT-D1*, with reduced activity of *FT1* contributing to the lower expression of *CAT-D1* in *ppd-1* null lines. To investigate this question, we measured *CAT-D1* transcripts in lines that lack a functional copy of *FT-B1*; these lines flower significantly later than wild-type (Dixon *et al.*, 2018). *CAT-D1* was expressed comparably in leaves and developing inflorescences of *ft-B1* mutant lines relative to wild-type, indicating the lower levels of *CAT-D1* transcripts in *ppd-1* are not caused by reduced *FT1* expression (Fig. 3B). Together, these results show that *CAT-D1* expression responds to changes in daylength and is regulated by the major photoperiod response gene, *Ppd-1*, but not *FT1.*

### Functional analysis of *CAT1*

To investigate the function of *CAT-1* in wheat, we predicted the structure of CAT-D1 using AlphaFold2 as implemented in ColabFold v1.5.5 (Mirdita *et al.*, 2022). Using the DALI structure comparison server (Holm, 2020), we identified *Gk*ApcT, an amino acid polyamine and choline transporter from *Geobacillus kaustophilus*, to be the closest experimentally determined structure to CAT-D1. Superimposition of the predicted CAT-D1 structure on the structure of *Gk*ApcT yields a root mean square deviation of 1.03 Å, indicating a high degree of structural similarity (Jungnickel *et al.*, 2018).

**Fig. 4.**
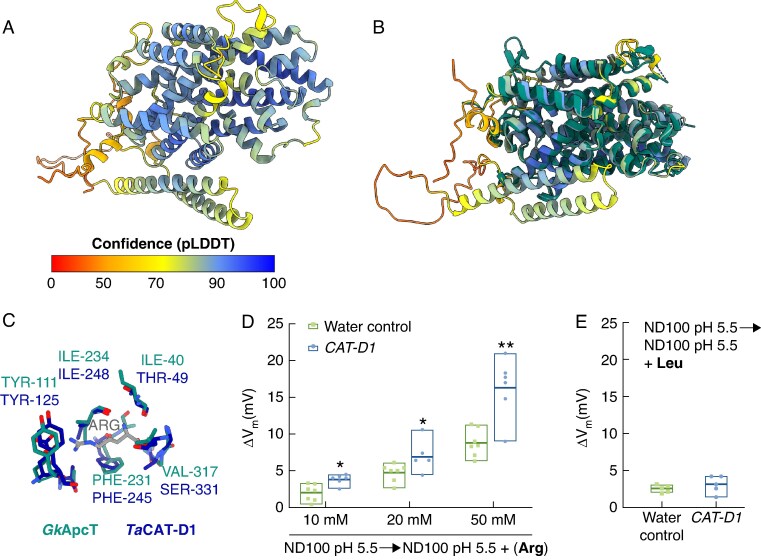
Predicted structure of CAT-D1 protein and analysis of arginine transport. (A) AlphaFold2 (AF2) prediction of CAT-D1. The CAT-D1 model is coloured according to prediction confidence, as represented by a predicted local-distance difference test (pLDDT) per residue. (B) AF2 model of CAT-D1 superimposed on the *Gk*ApcT structure with an root mean square deviation of 1.03 Å. (C) CAT-D1 (blue) maintains the necessary residues for arginine coordination, as determined from the experimentally derived model of *Gk*ApcT (green). (D) *Xenopus* oocytes expressing CAT-D1 protein (blue) transport arginine at increasingly higher amounts as arginine concentration rises in solution, relative to water control oocytes (green). (E) *CAT-D1*-injected oocytes do not transport leucine, when compared with the water control samples. Data are the average ± standard error of the mean of six biological replicates per reaction. **P < 0.01; *P < 0.05.

The predicted structure of CAT-D1 is formed of 14 transmembrane helices that are arranged in a conformation typical of the APC superfamily, which surrounds a gated channel (Fig. 4A–C). The predicted structure of CAT-D1 also demonstrates an amino acid binding site that shares conserved residues with that of *Gk*ApcT, including Phe245, Ala246 and Ile248 (homologous to Phe231, Ala232 and Ile234 of *Gk*ApcT), which interact with the carbonyl group of the incoming amino acid (Fig. 4C) (Jungnickel *et al.*, 2018).

To investigate transporter function of CAT-D1, we functionally characterized the protein using *Xenopus laevis* oocytes that were injected with *CAT-D1*-complementary RNA, relative to water controls (Fig. 4D). A two-electrode voltage-clamp electrophysiology assay showed that the resting membrane potential of *CAT-D1-*injected oocytes bathed in L-arginine shifted towards a positive current more significantly than that observed in water-injected oocytes, and the difference increased as the concentration of L-arginine was raised from 10 to 20 and 50 mm (Fig. 4D). Given L-arginine is positively charged at pH 5.5 ([H^+^] = 3.2 μm, the pH used in this experiment), a more significant positive shift in resting membrane potential indicates greater influx of L-arginine, thus indicating more L-arginine was transported into the *CAT-D1*-injected oocytes. Conversely, bathing *CAT-D1*-injected oocytes in 10 mM l-leucine did not induce a significant difference in the resting membrane potential relative to the water-injected oocytes (Fig. 4E). These results indicate CAT-D1 is a functional transporter of L-arginine.

### Genetic analysis of *CAT1* function in wheat

**Fig. 5.**
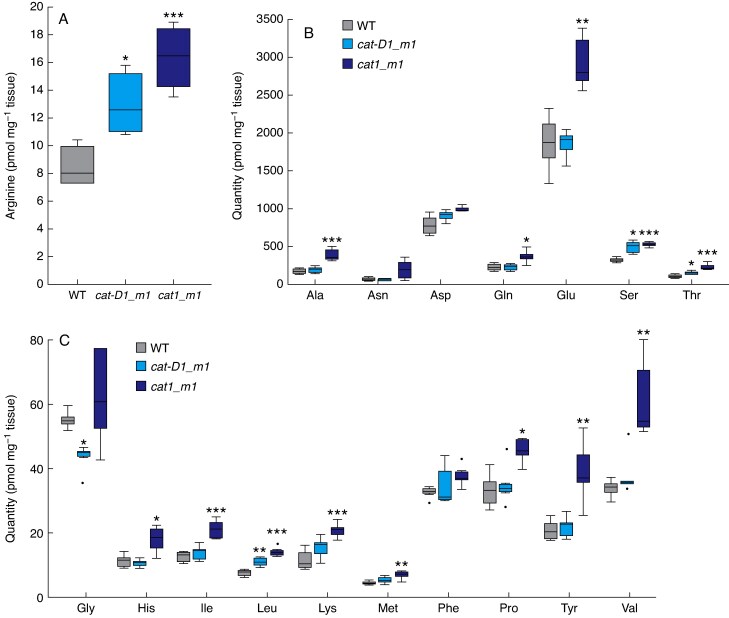
Amino acid analysis in *cat1_m1* lines. (A) Leaves of *cat-D1_m1* (light blue) and *cat1_m1* (dark blue) mutants contain more L-arginine than those from wild-type (grey). Analysis of the (B) abundant and (C) scarcer amino acids in leaves of *cat-D1_m1* (light blue) and *cat_m1* (dark blue) mutants relative to wild-type, from plants grown under long-day photoperiods. In the boxplots (A–C), each box is bounded by the lower and upper quartiles, the central bar represents the median, and whiskers indicate minimum and maximum values of six biological replicates. ***P < 0.001; **P < 0.01; *P < 0.05.

To further investigate the function of *CAT1* in wheat, we analysed *cat1* mutant lines. Our analysis exploited TILLING lines of an ethyl methanesulfonate-induced mutagenesis population generated in the background of cv. ‘Cadenza’, from which we identified lines that contained nonsense alleles of *CAT1* (Supplementary Data Fig. S3). There is no copy of *CAT-A1* in cv. ‘Cadenza’; however, quantitative transcript analysis showed that, unlike cv. ‘Paragon’, *CAT-B1* was expressed similarly to *CAT-D1* in ‘Cadenza’ (Supplementary Data Fig. S3). Our genetic analysis of *CAT1*, therefore included investigation of *cat-B1*/*cat-D1* double mutants (termed *cat1_m1*), which was complemented by investigation of a *cat-D1* single mutant (termed *cat-D1_m1*; Supplementary Data Fig. S3). As the *X. laevis* experiments indicated that CAT1 transports L-arginine, we asked if L-arginine levels were modified in the *cat-D1_m1* and *cat1_m1* plants, using plants grown under long-day photoperiods. There was a significant increase in L-arginine in leaves of *cat-D1_m1* and *cat1_m1* relative to wild-type (Fig. 5A). The increase in L-arginine was accompanied by a significant rise in levels of other amino acids, including serine, threonine and leucine, which were higher in both *cat-D1_m1* and *cat1_m1*, as well as glutamine, alanine, histidine, phenylalanine, tyrosine, valine, methionine, isoleucine and lysine, which were significantly higher in the *cat1_m1* double mutant (Fig. 5B, C).

**Fig. 6.**
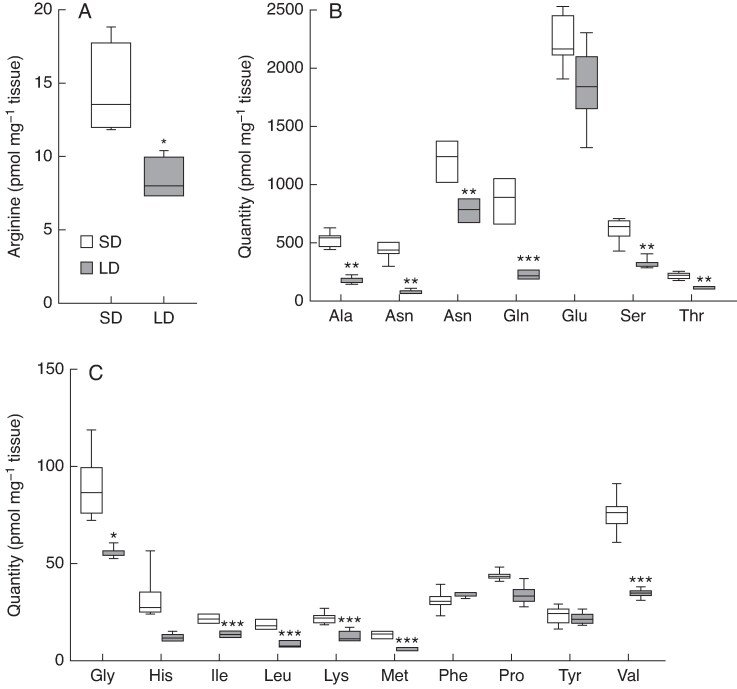
Photoperiod-dependent analysis of amino acids in wheat leaves. (A) Leaves of wheat plants grown under short-day photoperiods (SD, white) contain more L-arginine than those grown under long-day photoperiods (LD, grey). Quantification of (B) abundant and (C) scarce amino acids in leaves of plants grown in short days and long days. In the boxplots (A–C), each box is bounded by the lower and upper quartiles, the central bar represents the median, and whiskers indicate minimum and maximum values of five biological replicates. ***P < 0.001; **P < 0.01; *P < 0.05.

Given L-arginine levels increase in *cat-D1_m1* and *cat1_m1* mutants, and *CAT1* is expressed under long- but not short-day photoperiods, we hypothesized that L-arginine would be higher in wheat grown under short daylengths relative to plants grown under long photoperiods. To test this hypothesis, we grew wheat seedlings under short-day photoperiods (8 h light/16 h dark) until the four-leaf stage, and then shifted half of the plants to long daylengths (16 h/8 h) for 5 d before collecting leaf samples for amino acid quantification. We found that L-arginine levels were significantly higher in plants grown in short days relative to those shifted to long days (Fig. 6A). The lower levels of L-arginine were partnered with significant decreases in aspartate, asparagine, serine, glutamine, threonine, alanine, glycine, proline, valine, methionine, isoleucine, leucine and lysine (Fig. 6B, C). The accumulation of amino acids, including L-arginine, in leaves of plants grown under short-day photoperiods correlates positively with the rise in amino acids that occurs in the *cat1_m1* plants (Supplementary Data Fig. S4). Together, these results show that L-arginine accumulates in wheat leaves when *CAT1* is not expressed or does not encode a functional protein, and indicate that CAT1 influences the amino acid profile of plants grown under long-day photoperiods.

### Phenotypic analysis of *cat1* mutants

**Fig. 7.**
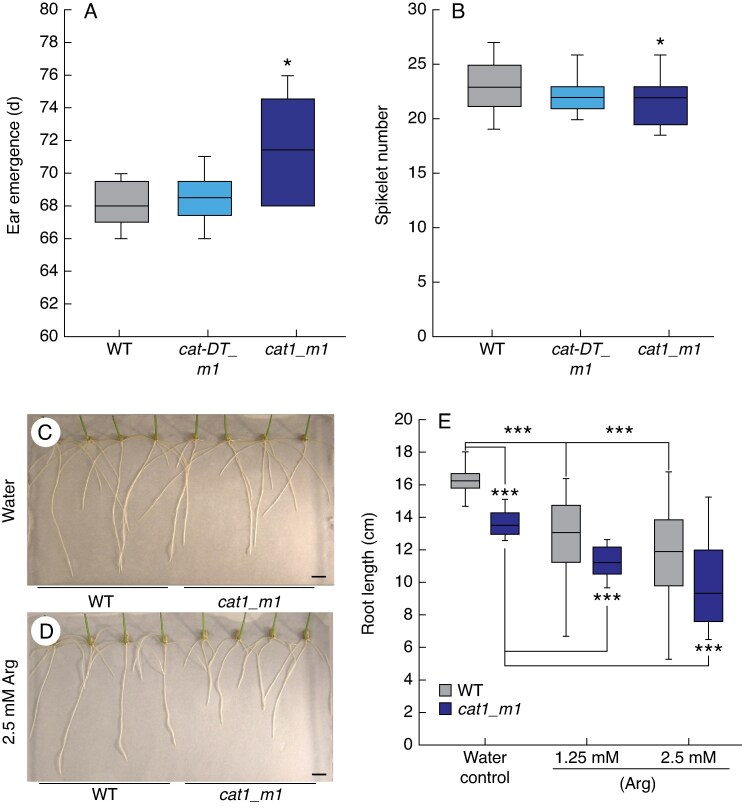
Phenotype analysis of *cat-D1_m1* and *cat1_m1* mutant lines. (A) Days to ear emergence for wild-type (WT, ‘Cadenza’), and *cat-D1_m1* and *cat1_m1* mutant lines under long-day photoperiods. (B) The *cat1_m1* double mutant plants produce significantly fewer spikelets than WT and *cat-D1_m1* mutants. (C, D) Roots of WT and *cat1_m1* mutants grown on (C) agar with water or on (D) agar that contains L-arginine (2.5 mm) at 7 d after germination (scale bar, 1 cm). (E) Roots of WT (grey) plants grow longer than those of *cat1_m1* double mutants (blue) at 7 d after germination, and arginine treatment (two different concentrations) reduces root growth in both WT and *cat1_m1* double mutants. In the boxplots, each box is bounded by the lower and upper quartiles, the central bar represents the median, and whiskers indicate minimum and maximum values of 6 (A–B) or 10–12 (E) biological replicates. ***P < 0.001; *P < 0.05.

Given *Ppd-1* influences *CAT1* expression and key reproductive traits of wheat, including phenology and inflorescence architecture, we asked if flowering time and spikelet number are modified in *cat-D1_m1* and *cat1_m1* relative to wild-type. Under long-day photoperiods, *cat-D1_m1* plants flowered at the same time as wild-type (68 d after germination), while *cat1_m1* flowered slightly but significantly later than wild-type (71 days after germination; Fig. 7A). The *cat1_m1* mutants produced significantly fewer spikelets than wild-type (21.4 ± 0.8 vs 23.3 ± 0.8; *P* = 0.019); spikelet number was lower, but not significantly so, in the *cat-D1_m1* mutant (Fig. 7B). These results indicate that CAT1 influences key reproductive development traits of wheat, which is consistent with *CAT1* expression being promoted by *Ppd-1* and by exposure to inductive long-day photoperiods.

As the transcript analysis showed that *CAT1* is expressed to similar levels in roots as in developing inflorescences of cv. ‘Cadenza’ (Supplementary Data Fig. S3), we asked whether root growth is altered in the *cat1_m1* mutants. Seedlings of *cat1_m1* mutants were grown on agar, and root length was measured 7 and 10 d after germination. The *cat1_m1* mutants produced roots that were significantly shorter than wild-type at both 7 d (13.59 ± 0.24 vs 16.35 ± 0.32) and 10 d (20.41 ± 0.32 vs 23.73 ± 0.27) post-germination (Fig. 7C, E; Supplementary Data Fig. S5). Given L-arginine accumulated to higher levels in *cat1* mutants relative to wild-type, we then asked whether L-arginine treatment influenced root growth by germinating seedlings on agar that had been supplemented with L-arginine. Wild-type seedlings grown on agar with L-arginine (1.25 and 2.5 mm) produced significantly shorter roots than those grown on the control media at both time points, with root growth being progressively inhibited by increasing L-arginine concentrations (Fig. 7C–E; Supplementary Data Fig. S5). The root length of the L-arginine-treated wild-type plants was like those of the control *cat1_m1* plants, which is consistent with L-arginine suppressing growth and *cat1_m1* mutants containing more L-arginine (Fig. 7E). Root growth of the *cat1_m1* mutants was also restricted by L-arginine treatment, with length reducing sequentially with higher concentrations of L-arginine (Fig. 7E). Based on these results, we conclude that *CAT1* contributes positively to wheat root growth during seedling stages, which is inhibited by treatment with L-arginine.

## DISCUSSION

Our analysis of *CAT1* indicates it is a photoperiod-responsive gene in wheat that encodes a protein capable of transporting arginine. The photoperiod responsiveness of *CAT1* expression is supported genetically by our analysis of lines containing null alleles of the major photoperiod sensitivity gene, *Ppd-1*. We found that *CAT-D1* is not expressed in leaves or inflorescence meristems of plants that do not contain functional alleles for the three homeologues of *Ppd-1*, indicating *Ppd-1* is required for *CAT1* expression in wheat. This discovery extends our understanding of Ppd-1 function, which is largely restricted to its control of flowering time and inflorescence architecture, and provides insights into genetic and environmental factors that influence expression of cation amino acid transporters (Beales *et al.*, 2007; Boden *et al.*, 2015; Ochagavia *et al.*, 2018; Perez-Gianmarco *et al.*, 2019; Gauley and Boden, 2021; Gauley *et al.*, 2024). A potential role for Ppd-1 in controlling the expression of genes that influence amino acid transport and metabolism is consistent with metabolite profiling performed in *Arabidopsis* mutants that lack functional copies of three pseudo-response regulators (*PRR5*, *PRR7* and *PRR9*) that are homologous to *Ppd-1*; analysis showed that levels of multiple primary metabolites, including amino acids such as arginine, were modified significantly in the *prr5*/*prr7*/*prr9* mutant line (Fukushima *et al.*, 2009). Our analysis of wheat *CAT* genes indicates the influence of *Ppd-1* is limited to *CAT1*; however, further transcriptome and gene network analyses in leaf and root tissues of the photoperiod-insensitive and *ppd-1* null lines may show that *Ppd-1* and daylength have broader roles in influencing the activity of genes encoding amino acid transporters. To explore this possibility, it would be valuable to test if *Ppd-1* functions as a transcription factor that directly regulates the expression of both amino acid transporters and flowering-time genes. A broader role for photoperiod in controlling amino acid metabolism or distribution is supported by levels of multiple amino acids, including arginine and lysine, reducing in leaves when plants were transferred from short- to long-day conditions.

Our transcript analysis showed that *CAT1*, along with *CAT2*, *CAT5*, *CAT6*, *CAT8* and *CAT11*, is expressed within the developing inflorescence, indicating that CATs contribute to flowering and spikelet/floret formation in wheat. The expression of *CAT* genes in developing inflorescences is consistent with the strong expression of *CAT1*, *CAT2*, *CAT5*, *CAT6* and *CAT9* in reproductive tissues (flowers and developing siliques) of *Arabidopsis* (Su *et al.*, 2004; Hammes *et al.*, 2006; Yang *et al.*, 2014, 2015). A potential role for CAT1 during early inflorescence development is supported by the *cat1* mutants producing fewer spikelets than wild-type, and with *CAT-D1* transcripts being present from the VG until the TS stage, which is when spikelet number is determined (Gauley and Boden, 2021). The late-flowering phenotype of *cat1* mutant plants suggests that CAT1 or arginine transport contributes to timing of flowering in wheat; interestingly, *Arabidopsis* plants that overexpress *CAT1* flowered earlier than wild-type when plants were grown under short daylengths, but no difference in flowering time was detected for null mutants (Yang *et al.*, 2014). A potential role for CAT1 during flowering is also supported by *CAT-B1* and *-D1* expression being induced in leaves as daylength extends from 10 to 11 h, which is consistent with the photoperiods that promote activity of the central integrator of flowering, *FLOWERING LOCUS T1* (Gauley and Boden, 2021).

The analysis of CAT1 transporter function and its expression throughout different wheat tissues indicate it contributes to amino acid movement in source and sink tissues. For example, our *Xenopus*-based assay and the predicted model of *CAT-D1* support a role for *CAT1* as an arginine transporter in wheat (Jungnickel *et al.*, 2018). This role is consistent with reports in other eukaryotes of CAT1 being a transporter of cationic amino acids, with yeast-based assays identifying *Arabidopsis* CAT1 as an arginine and histidine transporter, and roots from *CAT1* overexpressing plants taking up more lysine than those of wild-type controls (Frommer *et al.*, 1995; Yang *et al.*, 2014; Jungnickel *et al.*, 2018). Our expression analysis indicates CAT1 functions in both source and sink tissues, with *CAT-B1* and *-D1* transcripts detected in developing inflorescences, leaves and roots, and to a lesser degree in stems. Similarly, *CAT1* is expressed throughout multiple tissues in other plants, including roots, leaves, stems, floral organs and siliques in *Arabidopsis*, the hypocotyl, inflorescences, flowers and axillary meristems of soybean, and the roots and leaves of maize and rice (Su *et al.*, 2004; Zhao *et al.*, 2012; Yang *et al.*, 2014; Cheng *et al.*, 2016; Wan *et al.*, 2017; Islam *et al.*, 2024). Our analysis supports a potential role for CAT1 in roots of wheat, with root growth being restricted in *cat1_m1* mutants and by increasing concentrations of arginine.

In summary, our analysis indicates *CAT1* is a photoperiod-responsive gene that encodes an arginine-transporting protein that influences inflorescence development and root growth in wheat. These results suggest that *Ppd-1* has a broader role beyond regulating flowering time and spikelet architecture, which is linked to the remobilization of nitrogen-based assimilates from source to sink tissue during reproductive development. Our work, therefore, provides a platform to understand the processes linking inflorescence development and resource allocation, which are vital traits that influence the yield and quality of wheat grain.

## Supplementary Material

mcaf206_Supplementary_Data

## SUPPLEMENTARY DATA

Supplementary data are available at *Annals of Botany* online and consist of the following: Table S1: List of genes encoding CATs in bread wheat. Figure S1: Transcript analysis of *CAT* genes that are expressed in the developing inflorescence. Figure S2: *CAT-D1* expression is induced by long-day photoperiods. Figure S3: Expression analysis of *CAT1* in *cv.* Cadenza and development of nonsense mutant lines. Figure S4: Amino acid correlation analysis. Figure S5: The *cat1* mutants produce shorter roots than wild-type plants.
