# Supplementary material for: CATION AMINO ACID TRANSPORTER1 encodes an arginine transporter whose expression is influenced by daylength and Photoperiod-1
Source: Ann Bot. 2025 Sep 12;137(4):1001–12. doi: 10.1093/aob/mcaf206 (PMC13095878; doi:10.1093/aob/mcaf206)
Supplement: mcaf206_Supplementary_Data [file mcaf206_supplementary_data.pdf]

Table S1 – List of genes encoding cation amino acid transporters in bread wheat

Gene name	A genome homeologue	B genome homeologue	D genome homeologue
CAT1		TraesCS1B02G155400	TraesCS1D02G137900
CAT2	TraesCS2A02G390100	TraesCS2B02G409100	TraesCS2D02G388600
CAT3	TraesCS2A02G425919	TraesCS2B02G446235	TraesCS2D02G423900
CAT4	TraesCS3A02G133100	TraesCS3B02G166500	TraesCS3D02G149300
CAT5	TraesCS4A02G299800	TraesCS4B02G013800	TraesCS4D02G011700
CAT6	TraesCS5A02G126900	TraesCS5B02G126000	TraesCS5D02G134500
CAT7	TraesCS5A02G042900	TraesCS5B02G046500	TraesCS5D02G051400
CAT8	TraesCS5A02G025400	TraesCS5B02G023300	TraesCS5D02G031800
CAT9	TraesCS5A02G375600		TraesCS5D02G385125
CAT10	TraesCS5A02G256600	TraesCS5B02G256000	TraesCS5D02G265100
CAT11	TraesCS6A02G244400	TraesCS6B02G280000	TraesCS6D02G226700

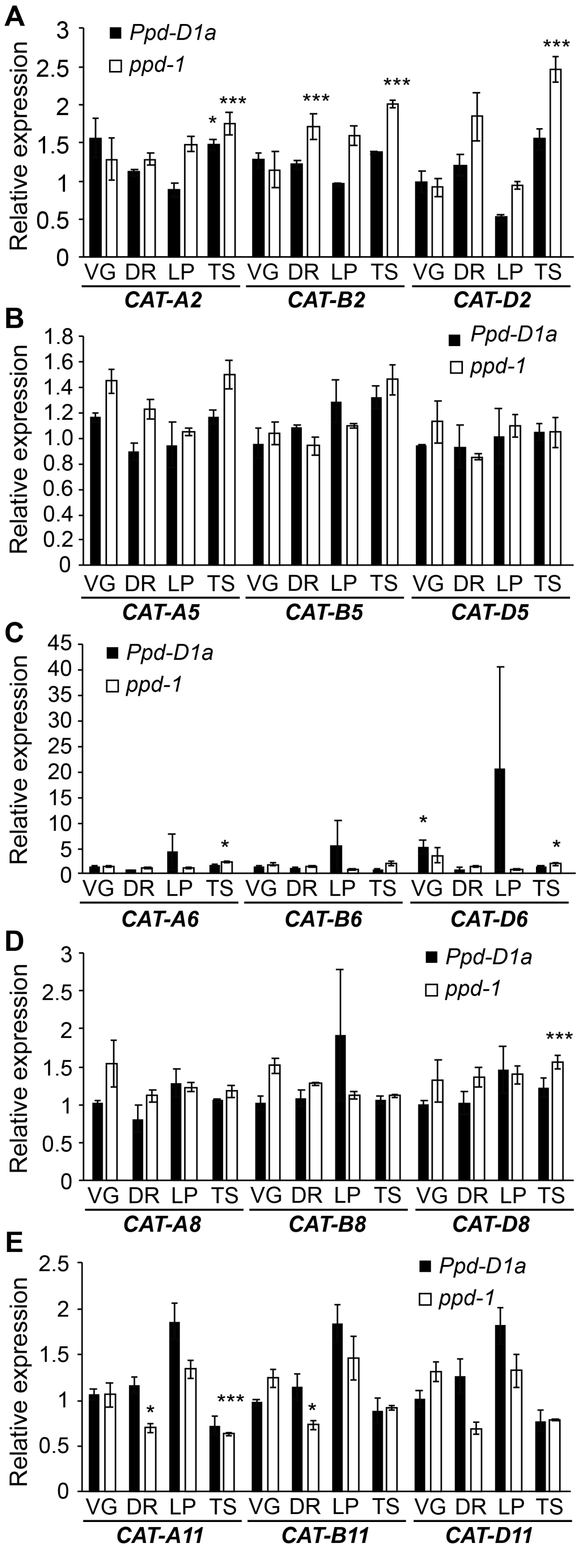

Supplementary Figure 1: Transcript analysis of *CAT* genes that are expressed in the developing inflorescence. Summary of relative transcript levels for *CAT* genes that are expressed in the developing inflorescence: (**A**) *CAT2*, (**B**) *CAT5*, (**C**) *CAT6*, (**D**) *CAT8*, and (**E**) *CAT11*. Values are shown for each homeolog at the vegetative (VG), double ridge (DR), lemma primordium (LP) and terminal spikelet (TS) stages. Expression values are shown for the *Ppd-D1a* photoperiod insensitive (black) and *ppd-1* null (white) lines, relative to wild-type (*cv.* Paragon). Data are the average ± S.E.M. of three biological replicates.

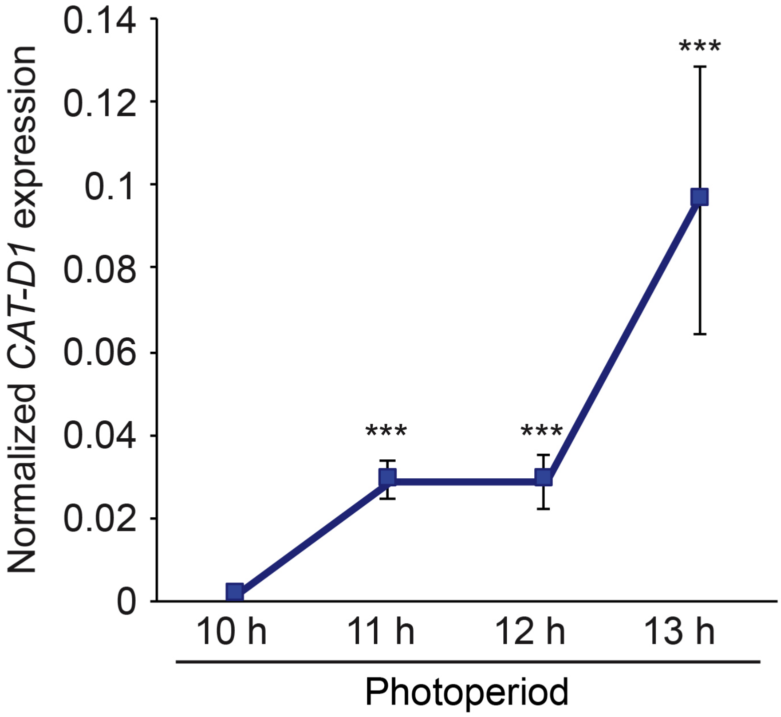

Supplementary Figure 2: *CAT-D1* expression is induced by long-day photoperiods. (**A**) *CAT-D1* is not expressed in leaves under short-day photoperiods (e.g., 10 h), and is induced as day-length increases. Values are the expression of *CAT-D1* at the timepoint of 16 h after dawn. Data is the average ± S.E.M of three biological replicates, ***$P < 0.001$.

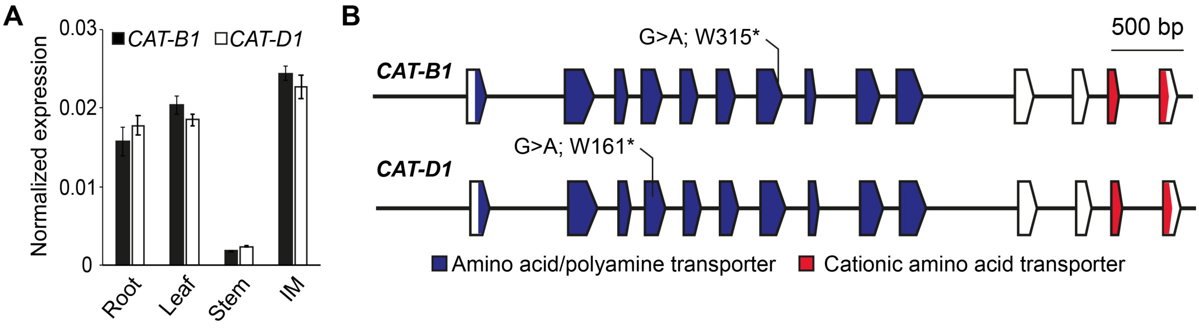

Supplementary Figure 3: Expression analysis of *CAT1* in *cv.* Cadenza and development of nonsense mutant lines. (**A**) The two homeologs of *CAT1*, *CAT-B1* (black) and *CAT-D1* (white), are expressed in roots, leaves, and inflorescence meristems of *cv.* Cadenza. Data is the average ± S.E.M of four biological replicates. (**B**) Gene structure of *CAT-B1* and *CAT-D1*, showing the position of premature stop-codon mutations used to generate nonsense mutant lines: *cat-D1_m1* and *cat1_m1* (double mutant). Exons are shown as arrows, and introns as black lines, with coloured arrows indicating regions of gene that encode conserved functional domains of the protein.

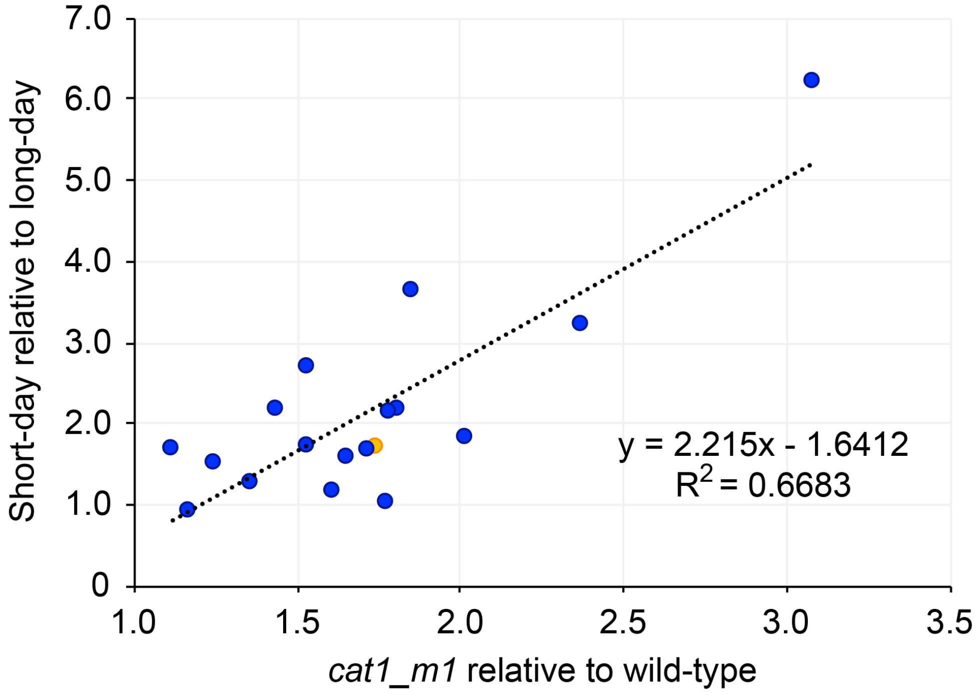

Supplementary Figure 4: Amino acid correlation analysis. The differences in amino acid levels under short-day and long-day photoperiods correlate positively with the changes in amino acids detected in *cat1_m1* mutants, relative to wild-type. Each data point (blue) refers to individual amino acids – *L*-arginine is represented by the orange dot.

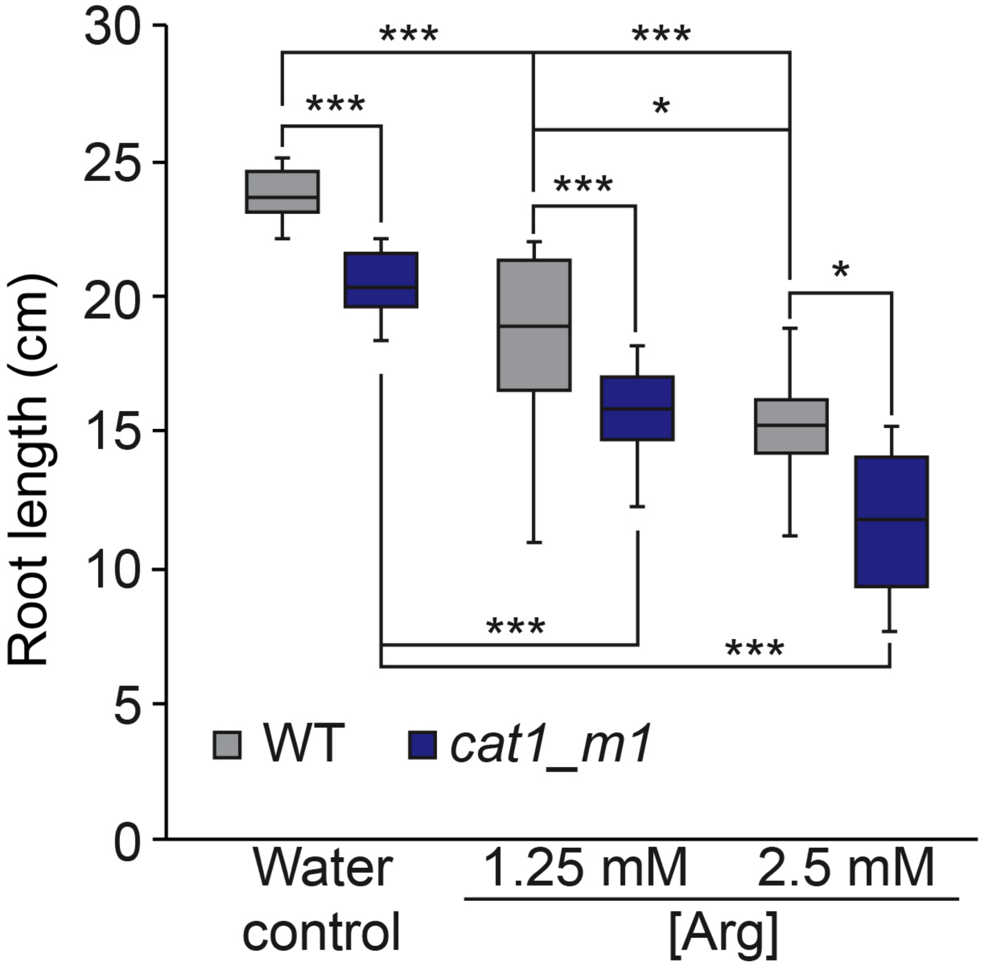

Supplementary Figure 5: The *cat1* mutants produce shorter roots than wild-type plants. Roots of wild-type (WT, grey) plants grow longer than those of *cat1_m1* mutants (blue) 10-days after germination, and arginine treatment (two different concentrations) reduces root growth in both WT and *cat1_m1* mutants. In the boxplot, each box is bounded by the lower and upper quartiles, the central bar represents the median, and whiskers indicate minimum and maximum values of 10-12 (**E**) biological replicates. ***$P < 0.001$; *$P < 0.05$.
